# Pontocerebellar Hypoplasia Type 1 and Associated Neuronopathies

**DOI:** 10.3390/genes16050585

**Published:** 2025-05-15

**Authors:** Mario Škarica, Gyula Acsadi, Sasha A. Živković

**Affiliations:** 1 Department of Psychiatry, Yale School of Medicine, New Haven, CT 06510, USA; mario.skarica@yale.edu; 2 Division of Neurology, Connecticut Children’s Medical Center, St. Hartford, CT 06106, USA; gacsadi@connecticutchildrens.org; 3 Department of Pediatrics, University of Connecticut School of Medicine, Farmington, CT 06030, USA; 4 Department of Neurology, University of Connecticut School of Medicine, Farmington, CT 06030, USA; 5 Department of Neurology and CMT Program at Yale, Yale School of Medicine, New Haven, CT 06510, USA

**Keywords:** pontocerebellar hypoplasia, hereditary motor neuropathy, hereditary motor-sensory neuropathy, spinal muscular atrophy, motor neuron disorder, mitochondrial dysfunction, RNA processing

## Abstract

Pontocerebellar hypoplasia is a rare neurodegenerative syndrome characterized by severe hypoplasia or atrophy of pons and cerebellum that may be associated with other brain malformations, microcephaly, optic nerve atrophy, dystonia, ataxia and neuromuscular disorders. At this time, there are 17 variants of PCH distinguished by clinical presentation and distinctive radiological and biochemical features in addition to pontine and cerebellar hypoplasia. PCH1 is defined as PCH variant associated with anterior horn degeneration in the spinal cord with muscle weakness and hypotonia, and is associated with recessive variants in genes VRK1, EXOSC3, EXOSC8, EXOSC9 and SLC25A46. Neuromuscular manifestations may clinically present as amyotrophic lateral sclerosis (ALS), motor neuropathy (HMN) or neuronopathy (non-5q spinal muscular atrophy; SMA) or sensorimotor polyneuropathy (HMSN). Physiologic functions of PCH1-associated genes include regulation of RNA metabolism, mitochondrial fission and neuronal migration. Overall, complex phenotypes associated with PCH1 gene variants ranging from PCH and related neurodevelopmental disorders combined with neuromuscular disorders to isolated neuromuscular disorders have variable outcomes with isolated neuromuscular disorders typically having later onset with better outcomes. Improved understanding of pathogenesis of pontocerebellar hypoplasia and its association with motor neuronopathies and peripheral neuropathies may provide us with valuable insights and lead to potential new therapeutic targets for neurodegenerative disorders.

## 1. Introduction

Pontocerebellar hypoplasia (PCH) is a rare neurodegenerative syndrome characterized by severe hypoplasia or atrophy of pons and cerebellum that may be associated with other brain malformations, microcephaly, optic nerve atrophy, dystonia, ataxia and neuromuscular disorders [1,2]. The exact overall incidence and prevalence of PCH are unknown. The incidence of one of its more frequent variants PCH2A was estimated at less than 1 in 200,000 [3]. The first classification of PCH by Barth in 1993. recognized 2 variants, PCH1 associated with spinal muscular atrophy and PCH2 associated with dystonia without anterior horn cell injury [4]. At this time, there are 17 variants of PCH distinguished by clinical presentation and distinctive radiological and biochemical features in addition to pontine and cerebellar hypoplasia [1,2]. PCH1 is defined as PCH variant associated with anterior horn degeneration in the spinal cord with muscle weakness and hypotonia [1,5]. The first detailed description of anterior horn cell disease with pontocerebellar hypoplasia in infants was provided by Goutieres in 1977, and since then recessive variants in five genes have been identified as causes of PCH1, including VRK1, EXOSC3, EXOSC8, EXOSC9 and SLC25A46 [1,5,6]. Earlier cases of infantile spinal amyotrophy with cerebellar abnormalities were considered as atypical cases of Werdnig-Hoffman disease [7]. Neuromuscular manifestations associated with variants of PCH1-related genes may clinically present as amyotrophic lateral sclerosis (ALS), motor neuropathy (HMN) or neuronopathy (non-5q spinal muscular atrophy; SMA) or sensorimotor polyneuropathy (HMSN) [1]. Electrodiagnostic testing (electromyography and nerve conduction studies are helpful to demonstrate peripheral nervous system abnormalities, and muscle biopsy typically shows neurogenic atrophy, although myopathic-like changes have been reported as well [8,9,10,11,12,13,14,15,16]. Physiologic functions of PCH1-associated genes include regulation of RNA metabolism, mitochondrial fission and neuronal migration, while genes associated with other PCH variants also play roles in tRNA splicing, RNA processing, regulation of GTP and selenocysteine synthesis and intracellular vesicle transport [1].

Typically, PCH manifests early with very limited or no developmental progress and poor survival [1]. In addition to early onset of neurodegenerative syndromes, recessive variants in PCH1-associated genes may also manifest in adulthood with isolated neuromuscular disorders. The age of onset is variable but the patients with motor and sensorimotor neuropathies without central nervous system abnormalities typically present much later than patients with fully manifesting PCH1 or combination of PCH1 and peripheral nerve disorders. In addition to biallelic autosomal recessive variants, PCH1 cases with compound heterozygosity have been reported as well [13,17].

We review clinical manifestations of gene variants associated with PCH1 and associated peripheral nervous system complications.

## 2. Genes

Genes associated with PCH1 variants include VRK1 (PCH1A), EXOSC3/EXOSC8/EXOSC9 (PCH1B-D) and SLC25A46 (PCH1E), and participate in regulation of neuronal migration, mRNA maturation and surveillance, and mitochondrial fission/fusion [1].

### 2.1. Vaccinia-Related Kinase 1 (VRK1; OMIM *602168)

The VRK1 gene (chromosome 14q32) encodes a serine/threonine kinase. VRK1 is highly expressed in and central and peripheral nervous system and in highly proliferative cells, such as those in testis, thymus and fetal liver. VRK1 participates in regulation of gene transcription, chromatin remodeling and DNA damage response as it interacts with histones and various transcription factors [18,19]. VRK1 interacts with multiple proteins including p53 and histones. Pathogenic variants of VRK1 have been associated with various neurodevelopmental and neuromuscular disorders, including pontocerebellar hypoplasia (PCH1A), microcephaly, motor and sensorimotor neuropathies and motor neuron disorders. With dominant expression in cell nucleus, it interacts with chromatin, several transcription factors and regulates several cellular processes, including the cell cycle, gene transcription, DNA damage responses, chromatin remodeling, and the assembly of Cajal bodies [18,20]. All VRK1 pathogenic variants are recessive—patients with clinical presentation are either homozygous or compound heterozygous. Functional analyses confirmed diminished levels of VRK1 protein and changes in RNA processing. VRK1 pathogenic variants are associated with pontocerebellar hypoplasia (PCH1A), microcephaly, SMA, ALS, HMN and HMSN.

### 2.2. Nuclear RNA Exosome Complex Components 3, 8 and 9 (EXOSC 3; OMIM *606489, EXOSC8; OMIM *606019, EXOSC9; OMIM *606180)

EXOSC3, EXOSC8, EXOSC9 genes (chromosomes 9p13.2, 13q13.3, 4q27, respectively) encode structural (non-catalytic component) subunits of RNA exosome, a multiunit ribonuclease complex. Eukaryotic RNA exosome is evolutionary conserved complex consisting of 10–111 subunits located in nucleus and cytosol and is crucial for both processing and degradation of a variety of RNAs, particularly in development and cell differentiation [21]. The exosome has a capacity to degrade any RNA but the activity of the isolated complex is weak. The activity is influenced by the cofactors and the RNA structures and it plays a role in controlling expression levels of specific mRNAs in response to environmental cues and during cell differentiation and development [21]. Variants of exosome component 3 (EXOSC3), exosome component 8 (EXOSC8) and exosome component 9 (EXOSC9) have all been associated with pontocerebellar hypoplasia type 1 (PCH1B-D). Clinical manifestations of EXOSC3/8/9 variants include PCH1B-D, microcephaly, optic atrophy, HMN and SMA.

### 2.3. Solute Carrier Family 25, Member 46 (SLC25A46; OMIM *610826)

SLC25A46 (chromosome 5q22.1) belongs to the SLC25 family of mitochondrial carrier proteins and is coded by nuclear genes. The proposed functions of SLC25A46 include facilitation of lipid transport across mitochondrial membrane at contact sites between mitochondria and endoplasmatic reticulum [22,23]. Mitochondrial fission is essential for mitochondrial division (proliferation) and the maintenance of its quality control [24]. SLC25A46 must be imported in inner mitochondrial membrane where it plays a role in mitochondrial network maintenance (fusion-fission balance) [23,24,25]. In autophagy, it is a part of mitochondrial-derived compartments (MDCs) which regulate the recycling of selective outer and inner mitochondrial membrane proteins (SLC25A46) within lysosomes [23]. In mammals, SLC25A46 facilitates lipid transport from endoplasmic reticulum to mitochondria in order to maintain mitochondrial cristae [26,27]. In yeast overexpression systems, SLC25A46 interacts with mitofusin 2 (MFN2) and optic atrophy 1 (Opa1) genes which are associated with CMT2A (MFN2) and optic atrophy (Opa1) [22,23,25]. Variants in SLC25A46 are associated with PCH1E, and other manifestations include HMSN, optic atrophy, ataxia and Leigh syndrome [26]. Loss of function of SLC25A46 is associated with lethal PCH, contrasting other variants causing non-lethal optic atrophy [28].

## 3. Clinical Phenotypes

### 3.1. Pontocerebellar Hypoplasia Type 1 and Spinal Muscular Atrophy (PCH1-SMA)

In PCH1 associated with SMA, early onset of symptoms prenatally or at birth manifests with severe weakness and hypotonia, and these patients may die within first few months [8]. Later onset of symptoms is associated with prolonged survival ranging from 2 to 11 years, or well into their fifth decade [18,29,30]. Spectrum of other associated clinical symptoms includes polyhydramnios, congenital contractures, severe muscle hypotonia with respiratory failure, spasticity, hearing and vision impairment and psychomotor retardation. In addition to pontocerebellar hypoplasia, neuroimaging studies may also show thinning of corpus callosum, cortical atrophy and delayed myelination [30]. Electrodiagnostic testing of infants is often challenging. Nerve conduction studies show reduced amplitudes of motor responses with normal sensory nerve conduction studies (or may be normal), and needle electromyography reveals neurogenic chronic motor unit changes. If performed, muscle biopsies typically show neurogenic muscle atrophy. Neuromuscular symptoms include weakness leading to ambulation loss and even respiratory failure. PCH variants associated with motor neuron degeneration have been reported with variants of all genes associated with PCH1 (VRK1, EXOSC3/EXOSC8/EXOSC9, SLC2A46) (Table 1) [1].

### 3.2. Hereditary Motor Neuropathy and Motor Neuron Disorders Without Neurodevelopmental Abnormalities

Variants of PCH1-associated genes have been recognized as some of potential causes of non-5Q-SMA and other motor nerve/neuron disorders in the absence of neurodevelopmental abnormalities [31]. Hereditary motor neuropathies and non-5Q SMA are similar neuromuscular disorders with overlapping phenotypes manifesting with hyporeflexia, muscle weakness and without sensory symptoms [31]. Nerve conduction studies show reduced amplitudes of motor responses with normal sensory nerve conduction studies (or may be normal), and needle electromyography reveals neurogenic chronic motor unit changes. As in PCH1-SMA, muscle biopsies typical show chronic neurogenic changes. The onset of weakness may be distal or proximal, and respiratory muscles may be involved as well. Some patients with motor neuronopathies may exhibit signs of upper motor neuron dysfunction as well and are diagnosed with ALS [32]. Clinical course of VRK1-associated motor neuron disease may reveal very slow rate of progression with extended survival, even with childhood onset [32,33]. The onset of motor neuropathy may be early or late in the adulthood [15,16,29]. Motor neuron disorders and motor neuropathies without neurodevelopmental abnormalities have been reported with PCH1A (VRK1 gene) [10,16,32,34].

### 3.3. Sensorimotor Axonal Polyneuropathy with or Without Neurodevelopmental Abnormalities

There is a considerable overlap between axonal CMT (CMT2) and other neuromuscular and neurologic disorders and variants in same genes can manifest as either hereditary motor neuropathies, hereditary spastic paraparesis or hereditary axonal sensorimotor neuropathies (CMT2) [10,16,35]. Nerve conduction studies demonstrate sensory and motor abnormalities that would distinguish sensorimotor polyneuropathy from motor neuropathy and motor neuronopathies with exclusively motor abnormalities. Axonal sensorimotor neuropathy has been reported in patients with pontocerebellar hypoplasia and SLC25A46 variants (PCH1E), and may be overshadowed by other severe neurologic symptoms with early onset and short survival [28,30].

Variants of VRK1 may cause sensorimotor neuropathy without neurodevelopmental abnormalities with an onset after the age of 40 with a slow gradual progression [10,16,34]. Variants of SLC25A46 may present with sensorimotor polyneuropathy with or without neurodevelopmental abnormalities, and the absence of neurodevelopmental abnormalities usually indicates prolonged survival. However, both early mortality and extended survival have been reported with SLC5A46 variants with neurodevelopmental abnormalities.

## 4. Discussion

**Figure 1:**
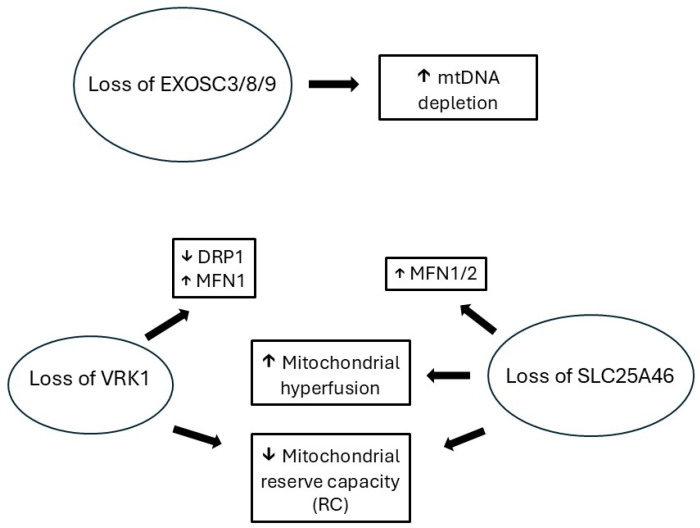
PCH1-associated genes and mitochondrial dysfunction. Legend: Loss of function of VRK1, EXOSC3/8/9 and SLC25A46 is associated with mitochondrial dysfunction manifesting with depletion of mitochondrial DNA, promotion of mitochondrial hyperfusion and loss of mitochondrial reserve capacity [22,41,42].

Variants of PCH1-associated genes can be associated with phenotypes ranging from severe neurodevelopmental disorders like PCH with or without neuromuscular and movement disorders, to motor neuron and peripheral nerve disorders without obvious signs of abnormal neurodevelopment. Neuromuscular phenotypes of variants of PCH1-associated genes belong to a wide spectrum of complex inherited neuropathies where peripheral nervous system disorders are only one of manifestations of complex clinical syndromes [36]. Early onset is typically associated with more severe disorders, especially with concurrent neurodevelopmental abnormalities. Later onset may be associated with slowly progressive milder syndromes and *forme fruste* manifestations, even in the presence of some neurodevelopmental abnormalities. Currently, our understanding of phenotype-genotype correlation in PCH-associated disorders and the impact of post-translational modifications on clinical manifestations remains limited. Recessive inheritance is required for all PCH1 variants, as either homozygous or compound heterozygous variants. Certain VRK1 variants (Arg 39 and Val236) seem to present with neurodevelopmental abnormalities only with homozygous variants, while compound heterozygous variants have only neuromuscular manifestations (Table 1). Nevertheless, due to paucity of cases, this may only represent a coincidence. Pathogenesis of PCH1 may follow two distinct molecular pathways associated with (a) destabilization of the protein leading to its depletion and (b) interference with its molecular actions (e.g., phosphorylation) as described with VRK1 and SLC25A46 variants [19,37]. In an experimental model of PCH1B in zebrafish, knockdown of EXOSC3 by antisense morpholinos led to a phenotype with a short curved spine and small brain. Co-treatment of zebrafish with wildtype EXOSC3 mRNA led to phenotype rescue [38]. Research studies have demonstrated interactions between various genes associated with PCH1 suggesting shared pathway mechanism(s), involving the dysfunction of cell cycle progression and mitochondrial dynamics (Figure 1) [18,21,24,37]. There is an overlap of pathophysiology of mechanisms leading to PCH1 and motor neuron disorders involving RNA processing and mitochondrial dysfunction which may potentially explain concomitant motor neuron injury manifesting as juvenile ALS, spinal muscular atrophy or hereditary motor neuropathy [39,40]. Laboratory studies showed evidence of overlapping patterns of mitochondrial dysfunction associated with PCH1A, PCH1B and PCH1E leading to increased mitochondrial hyperfusion, impaired mtDNA replication and metabolic glycolytic shift (Figure 1) [22,25,41,42]. The role of mitochondrial dysfunction in the degeneration of motor neurons in ALS has been proposed but its clinical significance and underlying mechanisms still remain unclear [43]. RNA processing abnormalities are associated with neurodevelopmental abnormalities and motor neuron disorders, and cytoplasmic aggregates of RNA-binding protein TDP43 are found across different variants of ALS [40].

Additionally, in some other PCH variants, rhabdomyolysis (PCH4) and weakness with hypotonia (PCH4 and PCH7) are also suggestive of neuromuscular involvement, but additional clinical information would be needed to characterize the involvement of peripheral nervous system [44,45,46].

Overall, complex phenotypes associated with PCH1 gene variants ranging from PCH and related neurodevelopmental disorders combined with neuromuscular disorders to isolated neuromuscular disorders have variable outcomes with isolated neuromuscular disorders typically having later onset with better outcomes (Table 1). The PCH1-related group of genes associated with pontocerebellar hypoplasia and peripheral nerve/motor neuron disorders shares physiologic roles of controlling cell cycle progression and mitochondrial dynamics (Figure 1). The connection between pontine and cerebellum development and motor neuron disorders is not well understood, but cerebellar atrophy has been demonstrated in various neurodegenerative conditions, including ALS [47]. Improved understanding of pathogenesis of pontocerebellar hypoplasia and its association with motor neuronopathies and peripheral neuropathies may provide us with valuable insights and lead to potential new therapeutic targets for neurodegenerative disorders. Currently, there is no treatment for PCH, including PCH1, but gene replacement therapies may be considered for these recessive disorders.

**Table 1:** Variants of PCH1-associated genes manifesting with neuromuscular disorders.

Gene	*Base Pair Mutation*	*AminoAcid Substitution*	Onset (Outcome)	Imaging/CNS Phenotype	NMD Phenotype	Reference
VRK1	c.197C>G (he); c.583T>G (he)	p.Ala66Gly; p.Leu195Val	Adult (alive at 48 yr)	Unk	SMA	[9]
	c.265C>T (he); c.769G>A (he)	p.Arg89*; p.Gly257Ser	Childhood (alive at 27 yr)	Normal	ALS	[32]
	C.266G>A (he); c.706G>A (he)	p.Arg89Gln; p.Val236Met	Infancy (alive at 9 and 10 yr)	Microcephaly	HMSN	[35]
	c.356A>G (he); c.961C>T(he)	p.His119Arg; p.Arg321Cys	Adult (alive at 32 yr)	Unk	ALS	[11]
	c.356A>G (he); c.1072C>T (he)	p.His119Arg; p.Arg 358*	Teenage (alive at 56 yr)	Brain atrophy	SMA	[34]
	c.398G>A (he); c.727G>A (he)	p.Arg133His; p.Asp243Asn	Teenage (alive at 28 and 33 yr)	Normal	HMN	[16]
	c.403G>A (he); c.583T>G (he)	p.Gly135Arg; p.Leu195Val	Childhood (alive at 20 yr)	Normal	ALS	[34]
	c.583T>G (he); c.701A>G (he)	p.Leu195Val; p.Asn234Ser	Adult (alive at 37 yr)	Unk	SMA	[9]
	c.607C>T (he); c.858G>T (he)	p.Arg203Trp; p.Met286Ile	Adult (alive at 59 yr)	Unk	SMA	[9]
	c.637T>C (ho)	p.Tyr213His	Childhood (alive at 35 yr)	Unk	HMSN	[48]
	c.706G>A (he); c.961C>T (he)	p.Val236Met; p.Arg321Cys	Adult (alive at 51 yr)	Normal	SMA	[15]
	c.710-14T>C (he); c.721C>T (he)	Intron; p.Arg241Cys	Childhood (alive at 28 yr)	Normal	ALS	[32]
	c.767C>T (he); c.800A>G (he)	p.Thr256Ile; p.Asp267Gly	Childhood (alive at 24 yr)	Normal ^#^	ALS	[33]
	c.788A>G (ho)	p.Asp263Gly	Childhood (alive at 25 and 29 yr)	Minimal cerebellar atrophy and normal	HSP	[49]
	c.961C>T (ho)	p.Arg321Cys	Adult, Teenage (alive at 55 yr)	Unk; Normal	HMN, SMA	[12,15]
	c.1072C>T (ho)	p.Arg358*	Antenatal (died at 9.5 and 11 yr) Toddler (alive at 10 yr)	PCH, microcephaly, Lissencephaly	SMA, HMSN	[30,35]
	c.1124G>A (ho)	p.Trp375*	Teenage (alive at 37, 42 and 46 yr)	Normal	SMA, HMN	[50,51]
	c.1159+1G>A (ho)	p.Arg387Hisfs*7	Infancy, Childhood (died at 13 yr, alive at 32 yr)	Normal	ALS, SMA	[52]
	c.1160G>A (ho)	p.Arg387His	Teenage (alive at 49 yr), Adult (alive at 59 and 61 yr)	Unk; Normal	HMSN; HMN	[10,16]
EXOSC3	c.92G>C (ho)	p.Gly31Ala	Infancy (died at 4 d–17 mo.)	PCH, microcephaly	SMA	[5,17]
	c.325-4_329dupGTAGTATGT (he); c.334G>A (he) c.395A>C (he)	p.Pro111*; p.Val112Ile; p.Asp132Ala	Infancy (died at 6 mo.)	PCH	SMA	[17]
	c.325T>A (he) c.395A>C (he)	p.Tyr109Asn; p.Asp132Ala	Infancy (died at 8.5 mo)	PCH	SMA	[17]
	c.395A>C (ho)	p.Asp132Ala	Infancy (died at 5 d–12 yr)	PCH, progressive microcephaly, cerebellar atrophy,	ALS, SMA	[17,38,42,53]
	c.395A>C (he); g.del37781240-37787410 (he)	p.Asp132Ala; intron. Deletion of exons 1–3	Infancy (died at 6 mo.)	PCH, optic atrophy	SMA	[17]
	c.404G>A (ho)	p.Gly135Glu	Infancy (died at 2 mo)	PCH	SMA	[17]
EXOSC8	c.238 G>A (ho)	p.Val80Ile	Birth (alive at 3 yr)	PCH, hypoplastic temporal lobes	SMA	[54]
	c.390+1delG (he); c.628C>T (he); c.815G>T (he)	p.Ser116LysfsTer27; p.Pro210Ser; p.Ser272Thr	Infant (alive at 16 yr)	PCH	SMA	[13]
	c.815 G>C (ho)	p.Ser272Thr	Infancy (died at 9–19 mo.)	PCH	SMA	[55]
EXOSC9	c.41T>C (ho)	p.Leu14Pro	Infant (died at 8–10 mo)	Cerebellar atrophy,	SMA, HMN	[56,57]
	c.41T>C (he); c.481C>T (he)	p.Leu14Pro; p.Arg161*	Infant (died at 10 mo.)	Cerebellar atrophy, CNS dysmyelination,	HMN	[57]
	c.151G>C (ho)	p.Gly51Arg	Infant (died at 2 mo.)	Cerebellar atrophy	SMA	[14]
	c.239T>G (he); c.484dupA (he)	p.Gly51Arg; p.Arg162Lysfs*3	Toddler (alive at 6 yr, died at 10 yr)	Cerebellar atrophy, normal pons	SMA	[14]
SLC25A46	c.42C>G (he); c.462+ 1G>A (he)	p.Tyr14*;(intron)	Infant (died at 1–18 d)	PCH	SMA	[8]
	c.165_165insC (he); c.746G>A (he),	p.[His56fs*94]; [Gly249Asp]	Childhood (alive at 43 yr)	Normal	HMSN	[22]
	c.413 T>G (ho)	p.Leu138Arg	Infant (alive at 15 yr)	Cerebellar atrophy, progressive myoclonic ataxia	HMSN	[58]
	c.1018C>T (ho)	p.Arg340Cys	Toddler (alive at 51yr)	Cerebellar atrophy	HMSN	[22,59]
	c.1022T>C (ho)	p.Leu341Pro	Infant (died at 2–6 wk)	PCH, optic atrophy, hypotonia, seizures	HMSN	[28]

ALS—amyotrophic lateral sclerosis; HMN—hereditary motor neuropathy; HMSN—hereditary motor-sensory neuropathy; PCH—pontocerebellar hypoplasia; SMA—spinal muscular atrophy; d—days; mo—months; wk—weeks; yr—years; unk—unknown; he—heterozygous; ho—homozygous; ^#^—incidental pineal cyst.
